# Social License and Animal Welfare: Developments from the Past Decade in Australia

**DOI:** 10.3390/ani10122237

**Published:** 2020-11-28

**Authors:** Jordan O. Hampton, Bidda Jones, Paul D. McGreevy

**Affiliations:** 1 Faculty of Veterinary and Agricultural Sciences, University of Melbourne, Parkville, Victoria 3052, Australia; 2 RSPCA Australia, P.O. Box 265, Deakin West, ACT 2600, Australia; bjones@rspca.org.au; 3 Sydney School of Veterinary Science, University of Sydney, Sydney, New South Wales 2006, Australia; paul.mcgreevy@sydney.edu.au

**Keywords:** sheep, cattle, dairy, greyhounds, horses, kangaroos, public perceptions, racing

## Abstract

**Simple Summary:**

“Social license to operate” (SLO) is the process by which a community grants or withholds permission to an industry to conduct its business. This article describes how animal welfare has recently become arguably the most crucial consideration underpinning SLO for Australian animal use industries in the past decade. Such industries include animal racing, wildlife harvesting, and the farming and live export of livestock. We posit that these industries are at risk of loss of SLO unless policies shift to proactive engagement with stakeholders and transparent monitoring of animal welfare outcomes.

**Abstract:**

“Social license to operate” (SLO) refers to the implicit process by which a community gives an industry approval to conduct its current business activities. It has become an important focus for many natural resource management fields (especially mining), but there is less awareness of its role in animal use industries. This article describes how animal welfare has recently become arguably the most crucial consideration underpinning the SLO for Australian animal use industries. It describes several industries in Australia that have faced animal welfare scrutiny in the past decade (2010–2020) to illustrate how persistent issues can erode SLO, lead to regulatory bans, and decimate previously profitable industries. Industries described include the live export of livestock, greyhound and horse racing, kangaroo harvesting, and dairy and sheep farming. In these cases, there has been intense public discourse but little scholarly progress. This article examines factors that may have contributed to these developments and suggests approaches that may assist these industries in maintaining their SLO. Animal welfare has become a mainstream societal concern in Australia, and effective management of the community’s expectations will be essential for the maintenance of SLO for many animal use industries.

## 1. Introduction

Australia has a particularly large diversity and scale of animal use industries [1]. Animal welfare issues arising in those industries have made the news headlines frequently in Australia over the past ten years. Industries with contentious animal practices have been embroiled in ongoing outrage as exposé investigations have emerged in the mainstream media [2]. Large established industries such as wool production, live export of livestock for slaughter, horse and greyhound racing, kangaroo harvesting, and dairy farming have been the target of hidden camera investigations and supporter-based advocacy campaigns from animal protection groups [2]. Concern about animal welfare practices is increasing and many animal use activities seem to be losing public support [1]. Response to community outrage has resulted in several temporary national or state bans on the operations of large industries, including live export of cattle in 2011 [3] and greyhound racing in 2017 [4]. 

This public reaction might be unexpected from a country that has traditionally been highly supportive of animal use industries. Australia is now a very different place to that depicted in folklore, having become a highly urbanized nation with a media-savvy population. In many cases, the source of the public outrage that triggered recent bans seems to have been the rising mainstream awareness of animal welfare driven by negative media content, including social media activism. The term used to describe loss of societal support through this process is loss of “social license to operate” (SLO). The concept gained particular currency in Australia when a government enquiry into the greyhound racing industry suggested that the industry had lost its SLO [5]. This commentary discusses the relevance of the SLO concept to animal welfare, using case studies from the past decade in Australia to show the outcomes of this phenomenon in high relief. This discussion is not prescriptive, but rather examines a contemporary phenomenon that is still evolving. 

## 2. Background 

SLO is a recently developed concept applied to an activity that has the ongoing but unstated approval of the local community and other stakeholders. Put another way, the SLO framework refers to the community tacitly giving an industry the right to conduct its current business, particularly to exploit publicly owned resources. Hence, SLO informally reflects prevailing public values and should not be confused with regulatory licenses. While the concept of SLO defies absolute definition [6], it has become an important focus for many natural resource management fields, including mining [7], energy production [8], fishing [9], and forestry [10]. There has traditionally been less awareness of its role in animal welfare, but there has been recent appreciation of its relevance to animal-based agriculture [11], wildlife use [12], animal racing [13], zoos [14], and hunting [15]. 

SLO can be tenuous, and if lost can decimate industries through loss of market access or legislative approval. Community outrage will inevitably lead to a loss of SLO in democracies, beginning with negative media and leading to political and regulatory bans. When public pressure, lobbying, and social media activism are strong enough, bans have been imposed by governments that are strongly affected by public perceptions in affluent and media-savvy jurisdictions. For example, the US state of California has long had a ban on Australian kangaroo product imports [16]. In addition, there are examples of loss of SLO for contentious activities without the implementation of legislation. Aside from governments, private companies may also decide to obstruct activities to which they are opposed. For example, in the wake of social media activity, multiple international airlines recently made the decision to refuse to transport hunting trophies [17,18]. 

## 3. Animal Welfare in Modern Australia

Since the 1990s, animal welfare has been steadily gaining importance in post-industrial countries [19,20]. This focus has been felt acutely in Australia, where animal welfare has become a core value of most of the population [1]. Animal welfare science scrutiny has expanded to nearly all animal use activities [21], and this renewed emphasis has been reflected in societal changes, such as expanded media coverage [20], increased prominence in the education of veterinarians [22], and legal amendments to increase maximum penalties for animal welfare offences [23]. There is an important distinction between animal welfare science and ethics-based animal advocacy [24]. There has been growing ideological opposition to animal use from ethics frameworks that prioritize respect for animal rights [25] or those invoking virtue ethics [26]. With negligible commitment to the measurement or refinement of animal welfare outcomes, such groups have been responsible for animal activism, which has been focused on disruption of activities and campaigns to end industries [27]. Taken together, these developments have been reflected in the recent rise of veganism and increasing availability of plant-based food products [28].

Combined with a highly urbanized, social-media-savvy population, Australia has consumers who, despite little first-hand knowledge of rural animal management practices [1], are generally well-informed. Animal welfare has become critical for the maintenance of SLO for animal-based industries [29] and poorly addressed concerns have led to the erosion of SLO for several practices [12]. Examples of such industries have spanned agriculture, animal racing, and wildlife use, and are discussed below. All of the industries discussed have experienced some degree of erosion of SLO in recent years, but the degree of public outrage and the way they have responded has varied markedly. 

## 4. Australian Animal Industries Having Experienced Recent Erosion of Social License

There are several Australian animal industries that appear to have tenuous SLO and could be vulnerable to public outrage from media coverage of practices that have had comparatively low levels of public awareness [30]. The following passages illustrate how industries have suffered from a loss of SLO.

### 4.1. Live Export of Sheep and Cattle

The Australian live export industry is the largest in the world, with millions of sheep and cattle having been exported via sea transport since 1985 [31]. Despite its scale and economic importance [32], the animal welfare implications of the industry have been under public scrutiny for decades [33]. Animal welfare issues that have created public outrage have included high stocking densities [34] and heat stress [35] during voyages and poor handling and slaughter methods used in destination countries [36]. In recent years, a series of media exposés have served to erode the industry’s SLO [2,37]. Following a 2018 television exposé, public support for the live export industry transiently declined in Australia [2], but a more recent study found little longer-term impact of this media event on public opinions of the red meat industry more broadly [38]. In the case of each exposé and the media attention it brought, the industry has faced trade suspensions [39] or has been forced to adapt rapidly to new regulations [40]. The industry has also embarked on an extensive public relations campaign, the focus of which is much less on the exporters and much more on farmers who supply the industry, advocating directly to the community [41]. SLO remains precarious for live export, with ongoing disagreement between the industry and the community regarding the animal welfare monitoring measures that should be undertaken and the degree to which these data should be made public [42].

### 4.2. Greyhound Racing

The Australian greyhound racing industry has faced an imperiled SLO in recent years over animal welfare issues [43,44]. There have been long-term concerns for the welfare of racing greyhounds [45] in Australia but little published animal welfare science or transparent monitoring of practices. Animal welfare concerns for greyhound racing include injuries to racing animals [46], euthanasia of surplus animals [47], and of most recent prominence, the illegal practice of live baiting (the use of live animals in training to induce greyhounds to chase the lure during races [48]). Public awareness of these issues rapidly expanded in 2015 when a television exposé revealed evidence of illegal and supposedly discontinued practices, including live baiting [4], suggesting that the industry had been practicing secrecy and deception. Greyhound racing currently faces an uncertain future in Australia because of erosion of its SLO, not least in the light of how these animal welfare issues have been handled [43], culminating in the imposition of bans on the industry (in one case subsequently reversed) in two Australian jurisdictions. 

### 4.3. Commercial Kangaroo Harvesting

Australian kangaroo (*Macropus* and *Osphranter* spp.) harvesting has faced intense animal welfare scrutiny, as well as determined opposition from animal rights activists and animal advocates opposed to wildlife killing [49]. Highly effective advocacy arising from these concerns, including documentary movies [50], has led to bans on the importation of kangaroo products in places such as California [16] and the possibility of the industry facing new potential bans in other export markets (e.g., China) [51]. The enduring animal welfare concerns associated with harvesting [52] include the frequency of adverse animal welfare events such as non-fatal wounding [53], as well as the manner of euthanasia of dependent juveniles (pouch young [54] and young-at-foot [55]). Despite these concerns, there is no transparent monitoring and limited auditing or inspection of animal welfare harvesting operations [56], resulting in ongoing uncertainty among consumers about the implications of consuming kangaroo products. The industry has shown some signs of proactivity, with a partial move to male-only harvest [52] being spurred by a realization that SLO was being lost primarily because harvesting females brings with it the killing or orphaning of juvenile kangaroos and pouch young [55]. Despite these challenges, it has been argued that commercial kangaroo harvesting has some of the best animal welfare outcomes of any large-scale meat production system in the world [57]. 

### 4.4. Horse Racing

Threats to SLO have recently been flagged for Australian horse racing [58], as they have for other Australian animal racing industries (e.g., greyhounds) [43], as well as for horse racing overseas (e.g., in the US) [13]. Some of the animal welfare issues relevant to Australian horse racing include mortalities during racing [59], so-called wastage (euthanasia of horses deemed unviable for racing [60], confinement [61], gastric ulcers [62]), whips [63], and tongue ties [64]. There are graphic animal welfare issues that are likely to cause public outrage (e.g., on-track mortalities and abattoir footage), as well as “sleeper” issues that are less likely to attract media attention (e.g., whips and wastage). The two Australian horse racing codes (harness racing and galloping) have made some improvements to the animal welfare issues they face, however reform has been slow compared to racing industries in some other countries, notably Norway [58]. There are many areas in which the horse racing industry could make strides in its journey towards what has been dubbed “ethical racing” [58], and thus could strengthen its SLO. 

### 4.5. Dairy Farming

The concept of a “social license to farm” [1] revolves around animal welfare issues, together with issues relating to climate change, water scarcity, environmental degradation, and declining biodiversity. Of all farming enterprises, dairy farming has faced perhaps the most intense animal welfare scrutiny, due to the nature of animal management, especially on large-scale farms. Increasing public concern has been observable for practices such as dairy calf management [65], cow–calf separation [66], calving induction, lameness management [67], and disbudding or dehorning of calves [68]. Currently, the most contentious animal welfare issues for Australian dairy include “mega dairies” (housing 700 or more animals) [69] and the bobby calf trade [70]. Dairy farming entails many inherent welfare problems, but also has the advantage that many consumers feel a reliance on their products, in a way that few consumers rely on meat or the racing industry to sustain their family. The dairy industry has also arguably displayed more proactivity in addressing animal welfare concerns than other animal use industries, with a long scientific tradition examining issues that affect cattle welfare and productivity [71,72,73]. 

### 4.6. Sheep Farming

“Social license to farm” [1] is also relevant to the interactions between animal welfare and Australian sheep farming. Practices used in contemporary Australian sheep farming have been under scrutiny over the past decade, and animal welfare concerns associated with contentious practices have also eroded demand for Australian wool. By far the most scrutinized practice in sheep farming has been “mulesing”, a surgical procedure that removes wool-bearing skin from the tail and breech area of sheep to prevent flystrike (cutaneous myiasis) [74]. Mulesing has attracted considerable adverse publicity. There was considerable media reporting of calls by People for the Ethical Treatment of Animals (PETA) to boycott the purchase of Australian wool, as well as instances where buyers have done so [1]. The industry responded in 2004 by committing to a phase-out of the practice by 2010, however this commitment was revoked [75] and mulesing continues to be used in Australia, albeit at slightly lower levels now that alternative flystrike remedies such as early wool-clipping and regular spraying are being adopted. In contrast, New Zealand officially banned mulesing in 2018 [1].

## 5. Why Have Some Australian Industries Experienced an Erosion of SLO?

With several large and historically well-supported animal industries currently facing loss of SLO and market access in Australia, the obvious question to ask is why has this situation arisen? There is not one simple answer to this question, but some overarching themes seem relevant to all case studies presented here. Factors that may have contributed to SLO loss in Australian animal industries include interaction with animal welfare science, engagement with stakeholders, transparency of animal welfare reporting, reliance on public relations, and the role of media. 

### 5.1. Interaction with Science

The role of animal welfare science in animal controversies is pivotal. In the subject areas outlined above, there has been intense public discourse on animal welfare issues but variable contributions of published science. In the case of dairy farming, there has been long-running engagement with science, however relatively little has occurred in the case of greyhound racing. Any commentary on SLO should clarify what is involved in the evaluation of an animal’s welfare and articulate the challenges associated with making reliable judgements. However, reaching scientific consensus on animal welfare issues is rarely straightforward and disparate opinions on the animal welfare implications of contentious practices have arisen through dissimilarities in values (e.g., the relative importance of animal health vs. natural behavior [76]). There are other challenges for science in the current age, with the unprecedented pace at which many of these crises have developed making meaningful application of animal welfare science problematic before contentious practices have been banned or voluntarily discontinued [9]. Nonetheless, the general absence of proactive animal welfare studies may have contributed to perceptions that industries such as kangaroo harvesting have not prioritized animal welfare. 

### 5.2. Stakeholder Engagement

Stakeholder engagement with groups other than scientists is another issue central to securing SLO. Not all animal use industries have taken progressive attitudes towards this process. Indeed, some Australian industries have taken a recalcitrant approach to animal welfare issues. Secrecy has been a popular approach for contentious industries in the past, however it appears to be losing popularity, increasingly attracting the mistrust of modern consumers and voters [77]. Secretive animal use practices have long been criticized by animal advocates who have referred to a ‘‘veil of secrecy’’ surrounding animal use [78]. Formerly secretive practices are increasingly being revealed by activism, facilitated by modern technologies (e.g., hidden camera investigations) [30]. The secrecy approach is exemplified by live export. For decades, the industry relied on secrecy during sea voyages and restricted animal welfare discussions regarding mortality rates. However, media exposé events changed the paradigm [2]. A similar story unfolded with greyhound racing, where supposedly discontinued practices (e.g., live baiting) were exposed by hidden-camera investigations [43]. Antagonism of critics rather than the establishment of constructive dialogue has also been seen in some Australian animal controversies. This amounts to the industry appearing to dismiss public concerns as mere reflections of a lack of community knowledge or understanding [1]. 

### 5.3. Transparency

Consumers and voters increasingly expect transparency from organizations that they are willing to trust [77]. When applied to animal welfare, transparency equates to public access to reliable data describing animal welfare outcomes. In the case of live export, commentators have suggested that an SLO for the continuance of the trade requires confidence in the transparency and validity of measures used to monitor animal welfare and regulate the industry [42]. This is an important distinction from prescribed inputs, intentions, language, or images [79]. For example, mortality rates and threshold levels for reporting have been implemented for live export of Australian sheep and cattle, however such reporting is absent in kangaroo harvesting. Some industries may resist calls for transparency due to fears of public awareness of actual outcomes or the cost of implantation. A lack of transparency about how animals are treated has been a common theme among many industries facing erosion of SLO [80]. The traditional resistant approach to transparency amounts to telling the public “we have the highest animal welfare standards and processes, however all of our outcomes are secret”. This approach relies on consumers missing the important difference between industries that say animal welfare is a priority and those that can demonstrate commitment to incremental improvements in the welfare of animals in their care [12]. 

### 5.4. Public Relations

In some cases, rather than examining or changing their practices, industries have seemed to rely exclusively on public relations when confronted with a SLO issue. In the past, many industries have attempted to deflect animal welfare concerns through the production of public relations materials attempting to portray a positive image. There is a critical distinction between attempts to improve animal welfare outcomes and attempts to improve public perceptions. The latter, namely reliance on public relations alone, can be defined as a monologue (that of the voice of the industry) that frames the issue, rather than a dialogue with stakeholders. Such one-way approaches to communication with stakeholders are usually formed without consultation [43]. Public relations strategies recognize that a threat to SLO exists, but attempt to address the threat with claims purported to ensure “humane” outcomes, although often lacking a science-based definition of what “humane” means [81]. The live export industry has been accused of this approach for much of its existence [82]. The reputational risks associated with a public relations strategy include the lack of support from animal welfare scientists and suspicion from consumers that animal welfare concerns may not be sincere.

### 5.5. The Role of Media

The media has played a central role in all the case studies discussed above, particularly in cases of opposition and activists attacking industries. Forms of opposition have include television exposés (live export [2] and greyhound racing [4]), documentary movies (kangaroo harvesting) [50], and social media attacks [15]. The recent history of animal welfare outrage in Australia has typically shown that media events, for example filming of poor outcomes in abattoirs or on live export ships, have driven expressions of public concern [1]. In some extreme cases, the impact of strong journalism broadcasts on free-to-air TV has seemingly eroded SLO virtually overnight [2]. Such exposés seem to be a hallmark of erosion of SLO in this context. This type of media event has often been associated with well-organized and popular advocacy campaigns to ban contentious animal practices. Abundant information regarding adverse animal welfare events in written reports or scientific papers does not seem to have imperiled SLO in the same way. However, public perception studies have indicated that in some cases, media coverage has little long-term impact on broader public attitudes, either because the message itself had little impact or because of lack of exposure to the media piece [38]. In some cases, such media advocacy has been successful in eroding public support and forcing legislators to take rapid action, such as temporary bans on the practices at the center of ongoing contention. Similarly, opposition expressed on social media can also exert rapid and effective pressure on policy makers and politicians [15].

## 6. Solutions and Challenges

Looking for current solutions to SLO issues is an ongoing challenge. The SLO framework is relatively new, and as with all newly developed theory, evidence linking societal opposition and policy change is currently limited [15]. Societal expectations are dynamic, especially in the field of animal welfare, so lasting one-stop solutions to SLO issues are unlikely to exist. Contentious practices such as live export and animal racing are likely to face increasing opposition in Australia in coming years. Industry responses to such challenges can be divided into broad-brush categories of proactive (industries that have owned their issues and embraced reform) and reactive (industries that have denied they have a problem and relied on public relations to restore their reputations) approaches [12]. While the reactive approach may have achieved short-term success in the past, it seems unlikely to appease most consumers in the long term. This suggests that proactive identification and mitigation of SLO threats is becoming essential for industries to be socially sustainable.

An established approach to securing SLO in this context is to recognize that community engagement is essential and to commit to the regular reporting of animal welfare metrics for practices that are currently contentious [12]. For practices that impose animal welfare impacts but currently lack widespread public concern, industries may proactively engage with animal welfare scientists before public awareness and outrage drive political intervention [21]. In the case of Australian industries, more published, publicly accessible animal welfare research undertaken by non-industry personnel is warranted. A feasible approach to facilitate this goal is to promote greater industry–university collaborations in welfare research [83]. This strategy relies on the premise that transparent demonstration of animal welfare outcomes, with a demonstrated commitment to improvement, will be more effective at maintaining public trust than secrecy [12]. This will certainly involve inconvenience and may threaten profit margins (at least in the short term), but ultimately will allow industries to continue to do business in the long term and may well lead to the opening of new markets. The costs of such reforms should be considered in the same vein as any other investment to sustain the industry and should be built into research and development budgets. This process ideally relies on engagement with independent scientists rather than in-house expertise. This is reflected in the Australian Productivity Commission recommending independence in setting animal welfare standards [84]. 

Solutions to established animal welfare problems will rarely be simple. The contemporary importance of animal welfare to the legitimacy of industries is worth considering within the nascent concept of “one welfare” [85], an extension of the “one health” concept that facilitates relatively more discussion of end-of life events. One welfare is underpinned by the premise that animal welfare depends on and influences human welfare and environmental sustainability. This is relevant in that amended practices designed to address a welfare issue may create others. For example, moving away from bobby calf transport in favor of on-farm killing may create undesirable environmental outcomes associated with the fate of calf carcasses [86]. In such situations, there is merit in industries applying a one welfare approach intended to addresses animal welfare, human welfare, and planetary health [85]. We do not wish to be prescriptive in discussing how industries should respond to contemporary animal welfare challenges, but rather we note that strategies emphasizing proactivity and transparency appear to have been largely successful in repairing and protecting SLO [12].

## 7. Conclusions

Animal welfare scrutiny is increasing for all industries. Proactivity in anticipating the effects of this scrutiny are central to the social sustainability of animal use industries. In the modern age, with increasing societal expectations of transparency, efforts to avoid scrutiny or attempts to deflect it through public relations alone are unlikely to be effective [12]. Furthermore, one welfare considerations are expected to take an increasingly important role in boardroom discussions that extend beyond animal welfare concerns in isolation. The concept of SLO appears to provide a useful framework for animal industries to build an improved model of consultation that engages the community in ways that could enhance transparency and build societal support [87]. Understanding stakeholder beliefs and desires will ultimately prompt industry to guide education, resolve pressing issues, and facilitate the regular reporting of incremental improvements in welfare outcomes [88]. In particular, there seems to be a need for industry–university collaborations to facilitate transparent animal welfare assessments. How the public perceives these animal welfare issues and how industries respond to them appear to be highly influential in shaping the market opportunities and long-term survival of affected industries. This trend seems to be especially prevalent in Australia. These issues are likely to affect other nations in the near future, and the case studies from Australia are instructive as to how different industry responses affect SLO. 
